# Comparing recruitment strategies to engage hard‐to‐reach men who have sex with men living with HIV with unsuppressed viral loads in four US cities: Results from HPTN 078

**DOI:** 10.1002/jia2.25798

**Published:** 2021-09-02

**Authors:** Chris Beyrer, Jowanna Malone, Stefan Baral, Zhe Wang, Carlos Del Rio, Kenneth H. Mayer, D. Scott Batey, Jason Farley, Theresa Gamble, Jill Stanton, James P. Hughes, Ethan Wilson, Risha Irvin, Oscar Guevara‐Perez, Adam Bocek, Josh Bruce, Ronald Gaston, Vanessa Cummings, Robert H. Remien, the HPTN 078 Study Team

**Affiliations:** ^1^ Department of Epidemiology Center for Public Health and Human Rights Johns Hopkins Bloomberg School of Public Health Baltimore Maryland USA; ^2^ StatisticalCenter for HIV/AIDS Research and Prevention Seattle Washington USA; ^3^ Department of Epidemiology Emory Center for AIDS Research Atlanta Georgia USA; ^4^ Beth Israel Deaconess Medical Center Harvard Medical School Boston Massachusetts USA; ^5^ The Fenway Institute Boston Massachusetts USA; ^6^ Department of Social Work University of Alabama at Birmingham Birmingham Alabama USA; ^7^ School of Nursing Johns Hopkins University Baltimore Maryland USA; ^8^ HPTN Leadership and Operations Center FHI 360 Durham North Carolina USA; ^9^ Department of Biostatistics University of Washington Seattle Washington USA; ^10^ Vaccine and Infectious Diseases Division Fred Hutchinson Cancer Research Center Seattle Washington USA; ^11^ Department of Pathology Johns Hopkins School of Medicine Baltimore Maryland USA; ^12^ HIV Center for Clinical and Behavioral Studies NY State Psychiatric Institute and Columbia University New York New York USA

**Keywords:** ARV, co‐infection, HIV epidemiology, men who have sex with men, recruitment, viral suppression

## Abstract

**Introduction:**

There is an urgent need to identify men who have sex with men (MSM) living with HIV with unsuppressed viral loads to prevent transmission. Though respondent‐driven sampling (RDS) is traditionally used for hard‐to‐reach populations, we compare how RDS and direct recruitment (DR) perform in identifying MSM living with HIV with unsuppressed viral loads and identifying MSM with socio‐demographics characteristic of hard‐to‐reach populations.

**Methods:**

This is a cross‐sectional analysis among 1305 MSM who were recruited from March 2016 to December 2017 for a case management intervention trial (HPTN 078). We recruited participants across four cities using RDS and DR methods: Birmingham, AL; Atlanta, GA; Baltimore, MD; and Boston, MA. Participants completed a socio‐demographic questionnaire and underwent HIV testing. We compare the proportion of MSM with HIV and unsuppressed viral loads (HIV RNA ≥ 1000 copies/ml) based on recruitment method using Pearson chi‐square tests. We also compare differences in race, income, healthcare coverage, education, sexual orientation, hidden sexuality and comfort with participating in the LGBT community between recruitment methods and perform non‐parametric trend tests to see how demographics change across RDS recruitment waves.

**Results:**

RDS recruited 721 men (55.2%) and DR yielded 584 men (44.8%). Overall, 69% were living with HIV, of whom 18% were not virally suppressed. HIV prevalence was higher among those recruited via DR (84%) compared to RDS (58%), *p* < 0.0001. Twenty per cent of DR recruits were not virally suppressed compared to 15% of RDS, though this was not significant. DR yielded a significantly higher proportion of Black participants and those with less than a high school diploma. The prevalence of low income, no healthcare coverage, bisexuality and hidden sexuality increased across RDS waves.

**Conclusions:**

DR was more efficient in identifying MSM living with HIV with unsuppressed viral loads; however, there was a higher proportion of hard‐to‐reach MSM who were low income, lacked health coverage, were bisexual and were not open with their sexuality in deeper waves of RDS. Researchers should consider supplementing RDS recruitment with DR efforts if aiming to identify MSM with unsuppressed viral loads via RDS.

## 1 INTRODUCTION

The HIV epidemic in the United States has disproportionately affected gay, bisexual and other men who have sex with men (MSM) since the first cases were identified in the 1980s [1]. In 2017, the US Centers for Disease Control and Prevention reported that 70% of all new infections among adults and adolescents in the United States were among MSM [2]. The US epidemic is also characterised by racial and ethnic disparities, with the highest HIV burdens occurring among Black MSM, followed by Latinx MSM [3, 4, 5, 6]. There is also a geographic component as HIV incidence rates among Black, Latinx and White MSM are all higher in the South and Southeast [7]. These racial health disparities among MSM are attributable to both individual level determinants such as low HIV testing rates and late presentation for HIV care, as well as network and structural level determinants such as lack of access to sufficient HIV care [3, 4]. Experiences of intersectional HIV and racial stigma among Black and Latinx MSM can also hinder access and sustained engagement with HIV care [8].

The HIV care continuum is a useful tool in characterising the journey of those living with HIV from being diagnosed to achieving and sustaining viral suppression, with linkage and engagement in care being critical to success [9, 10]. The treatment as prevention strategy calls to treat those living with HIV to achieve viral suppression and to prevent the spread of infection [11]. Thus, improving the identification and engagement of MSM living with HIV with unsuppressed viral loads is an urgent HIV research priority [12].

Finding MSM living with HIV who are virally unsuppressed can be difficult as this is a subset of an already 'hard‐to‐reach' population [13]. Additionally, MSM who are not virally suppressed can include those who remain untested and undiagnosed, who have an HIV diagnosis but have not initiated care, who have initiated care but have defaulted from treatment or who are on antiretroviral treatment (ART) but remain viremic from ART resistance and/or suboptimal adherence. Thus, the sampling frame for this population is not well understood and researchers should explore recruitment strategies that can effectively identify MSM who are not virally suppressed.

In the effort to identify MSM who are not virally suppressed, researchers must consider factors that contribute to a lack of engagement in research. For instance, MSM who lack health coverage or who do not openly identify as a sexual minority are likely to struggle with viral suppression [14], and are less likely to engage in research, [15] respectively. Black and Hispanic MSM as well as MSM of low socioeconomic status can be even harder to reach in HIV‐related studies due to mistrust of the medical community and low engagement in healthcare [16].

The HIV Prevention Trials Network (HPTN) 078 study used both respondent‐driven sampling (RDS) and direct recruitment (DR), to reach and identify MSM who were living with HIV and not virally suppressed for a case management intervention. This paper presents the findings from the recruitment phase of the study to determine which recruitment strategy resulted in a higher proportion of MSM who were not virally suppressed. We also assess any differences in socio‐demographics characteristic of hard‐to‐reach MSM: being Black or Hispanic, education lower than a high school diploma, low income, having no healthcare coverage, sexual minority status, hidden sexual orientation and reported discomfort in the LGBTQ community. We then explore if there is an increase in the proportion of MSM with these characteristics and the proportion of MSM who are not virally suppressed across increasing waves of RDS.

## 2 METHODS

### 2.1 Study sites

The study sites in HPTN 078 included Ponce de Leon Center Clinical Research Site (Atlanta, GA), Fenway Health Clinical Research Site (Boston, MA), Johns Hopkins University Clinical Research Site (Baltimore, MD,) and University of Alabama at Birmingham Clinical Research Site (Birmingham, AL). These cities were selected for the HPTN 078 protocol based on their high burden HIV epidemics among MSM.

### 2.2 Recruitment

Screening began in March 2016 and ended in December 2017. Participants were eligible if they were at least 16 years of age (for the Boston, MA, and Birmingham, AL, sites) or at least 18 years of age (for the Atlanta, GA, and Baltimore, MD, sites), assigned male sex at birth, and reported having anal sex with another man in the past 6 months.

Participants were recruited initially through RDS. RDS is a sampling method used in HIV surveillance and research to identify and recruit marginalised populations (including MSM, transgender women or people who inject drugs) [17, 18, 19, 20, 21]. It has also been a successful method in recruiting MSM of colour in the United States [22]. RDS relies on the identification and enumeration of a discrete number of 'seed' participants who are incentivised to refer members of their social or sexual networks to the study.

As it became challenging to use RDS alone to recruit MSM living with HIV who were not virally suppressed, we altered our approach by allowing the sites to directly recruit men presenting for HIV testing and with untreated HIV disease.

### 2.3 RDS recruitment procedures

The protocol team, which included an RDS expert (SB), reviewed the characteristics of the original seeds (∼8–10 per site) from each study site to identify racially and ethnically diverse individuals with expansive social networks. Each seed was given three coupons to disperse to social network peers. The coupons had an expiration date of approximately 2 weeks to encourage quick coupon return; however, sites were instructed to accept coupons past their expiration date until the end of data collection. When a potential participant returned with a coupon, they were screened for eligibility for the trial, and were offered three coupons to disperse to other MSM in their social network. Recruiters were reimbursed for each returned coupon brought back to the site by a potential participant. The reimbursement per coupon ranged from $15 to $30, as some study sites offered the same amount for all coupons, while others offered a higher amount for the first coupon, followed by lower amounts for subsequently returned coupons. Coupons were tracked using RDS Coupon Manager software version 3.0 [23] and a Microsoft Excel coupon log.

To encourage greater recruitment results, the RDS methodology was adapted over the course of the study. For example, originally only three coupons were distributed to all recruiters, but this was increased to six for those who were productive recruiters (i.e. those who had all three coupons returned or yielded at least one enrolment). Study staff also followed up with seeds and recruiters about unreturned coupons to further encourage distribution.

### 2.4 Direct recruitment procedures

DR began in October 2016, 7 months after the RDS began after consulting with community advisory boards on best practices in navigating DR. Study staff were asked to directly recruit for the study via targeted community testing events (e.g. testing in parking lots of gay clubs), partnerships with HIV testing agencies, clinics, hospitals, support groups, community‐based organisations and social media (e.g. by placing ads on Facebook and gay dating apps such as Grindr). Community testing events used rapid tests as per study site protocols, followed by referrals to clinics for men found to be living with HIV. Study sites used electronic medical records, partnerships with emergency rooms and new patient orientations to identify potentially eligible participants. Productive recruiters and enrolled participants were asked to informally refer people they thought might be eligible for the trial.

There were no differences in study procedures between participants recruited via RDS and DR, except that those recruited directly were not asked to distribute coupons to others unless they were invited to become a seed. Those who were invited to be seeds were still considered directly recruited, thus they are a part of the DR sample in this analysis (Table 1).

**Table 1:** Screening and enrolment by RDS and direct recruitment by study site

	Birmingham	Boston	Baltimore	Atlanta	Total
Total number of seeds	17	47	95	108	267
Number of coupons distributed	648	730	864	757	2999
Number coupons returned	205	210	245	139	799
RDS (number of coupons returned, eligible for screening and screened (all > Wave 0))	203	198	183	137	721
Direct recruitment (includes seeds)	263	90	98	133	584

Abbreviation: RDS, respondent‐driven sampling.

### 2.5 Key changes in recruitment procedures

In December 2016, we changed the protocol to allow transgender women to undergo screening as several had been referred by others during RDS recruitment and showed interest in participating, were assigned male at birth, and had anal sex with cisgender men within the last 6 months. However, we ultimately did not have a sizeable contingent of transgender women screened. In April 2017, we no longer required participants to have had anal sex within the prior 6 months to improve recruitment yield.

### 2.6 Data collection and measures of interest

#### 2.6.1 Socio‐demographics of hard‐to‐reach MSM

Once recruited, study staff determined if the participant was eligible for further screening. Participants then underwent informed consent for the screening visit. Upon screening, participants completed a computer‐assisted self‐interview in which they reported age, race/ethnicity, education level, income, whether they had health coverage (regardless of coverage type), sexual orientation (i.e. gay, bisexual or other), whether they were hiding their sexual orientation from others and whether they 'agree', 'disagree' or were 'neutral' to the statement 'Participating in the LGBT community is a positive thing'. Race/ethnicity was categorised as Black, Hispanic, White, Asian, Native American, Hawaiian Pacific Islander, Multiracial or Other. We dichotomised education level to compare those with less than a high school diploma to those with at least a high school diploma or beyond. We classified participants as low income if they reported less than $20,000 per year in annual earnings.

#### 2.6.2 HIV and viral suppression measurements

Participants also provided blood samples for HIV testing. Study staff conducted a rapid HIV antibody test (using oral fluid, fluid from a finger stick or whole blood sample) which if reactive was followed by confirmatory HIV testing that included a fourth‐generation assay. Study staff measured viral load among participants living with HIV. Participants were considered virally suppressed if their viral load was ≤1000 copies/ml as we believed this higher cut‐off (compared to <200 copies/ml) was more salient for transmission dynamics and excluded viral blips (i.e. short bouts of viremia that could exceed 200 copies/ml up to 500 copies/ml) [24].

### 2.7 Statistical analysis

We conducted Pearson chi‐square tests to detect any statistically significant comparisons between those recruited through RDS compared to those recruited through DR in Stata 15 [25]. Specifically, we aimed to determine if there would be a higher proportion of participants living with HIV and not virally suppressed recruited through RDS compared to among those recruited through DR. We also compare the breakdown of race, sexual orientation, education, income level, healthcare coverage, hidden sexual orientation and participation in the LGBT community between recruitment strategies to determine which strategy accrued MSM who were hard to reach. We subsequently conducted non‐parametric trend tests comparing the proportion of MSM with unsuppressed viral loads and MSM with hard‐to‐reach socio‐demographics across waves of RDS recruitment to determine if there were increases in these characteristics with increasing waves.

### 2.8 Ethical review

The local institutional review board for each study site approved the protocol prior to study implementation. Participants provided written informed consent prior to any study procedures. HPTN 078 is registered on ClinicalTrials.gov (NCT02663219).

## 3 RESULTS

### 3.1 Demographics

There were 1305 participants recruited for the study, with 721 (55.2%) recruited through RDS and 584 (44.8%) recruited through DR (Table 2). Among all participants, 902 (69%) were living with HIV, with 18% having unsuppressed viral loads (≥1000 copies/ml). Overall, 68% of participants were Black, 19% were White and 13% as another race. Twelve per cent of participants identified as Hispanic. The median age was 41 (inter‐quartile range (IQR): 30, 52). There were 15% with less than a high school diploma, 27% who were low income (<$20,000/year) and 16% who had no health coverage. Sixty‐six per cent identified as gay/lesbian/homosexual, 26% identified as bisexual and 8% identified as another sexual orientation. Fifty per cent reported hiding their sexual orientation from others and 21% disagreed with the statement 'Participating in the LGBT community is a positive thing to do'.

**Table 2:** Comparing socio‐demographics, HIV status and viral suppression by recruitment strategy

	Overall (*N* = 1305)^a^	RDS (*N* = 721)^a^	Direct recruitment (*N* = 584)^a^	Chi‐square test for group comparison
Characteristics	*N* (%)	*N* (%)	*N* (%)	*p*‐Value
Age (median, IQR)	41 (IQR: 30, 52)	41 (IQR: 29, 53)	40 (IQR: 31, 51)	0.79
Gender (self‐reported) Men Transgender women Cisgender women Other ender identity	1240 (95) 46 (4) 11 (1) 6 (<1)	695 (96) 17 (2) 6 (1) 2 (<1)	545 (93) 29 (5) 5 (1) 4 (<1)	0.10
Race Black White Asian Native American Hawaiian/Pacific Islander Multiracial Other	885 (68) 253 (19) 10 (1) 6 (0.5) 0 (0) 19 (1.5) 131 (10)	445 (62) 175 (24) 8 (1) 3 (0.4) 1 (0.1) 6 (0.8) 83 (12)	440 (75) 78 (13) 2 (0.3) 3 (0.5) 0 (0) 13 (2) 48 (8)	<0.0001
Ethnicity Hispanic Non‐Hispanic	153 (12) 1150 (88)	96 (13) 624 (87)	57 (10) 526 (90)	0.047
Education Less than high school diploma High school and beyond	192 (15) 1113 (85)	92 (13) 629 (87)	100 (17) 484 (83)	0.028
Income Low income (<$20,000) Higher income (≥$20,000)	347 (27) 953 (73)	189 (26) 531 (74)	158 (27) 422 (73)	0.705
Health coverage Yes No	1095 (84) 208 (16)	613 (85) 107 (15)	482 (83) 101 (17)	0.23
Sexual orientation Other Bisexual Gay/lesbian/homosexual	108 (8) 335 (26) 860 (66)	62 (9) 186 (26) 472 (66)	46 (8) 149 (26) 388 (67)	0.878
Hide sexual orientation from others Very much Somewhat Not at all	130 (11) 461 (39) 606 (51)	65 (10) 261(40) 328 (50)	65 (12) 200 (37) 278 (51)	0.383
Participating in the LGBT community is a positive thing Agree Neutral Disagree Prefer not to answer	677 (56) 280 (23) 248 (21) 98 (8)	377 (56) 159 (24) 133 (20) 51 (7)	300 (56) 121 (23) 115 (21) 47 (8)	0.803
HIV status Positive Negative	902 (69) 392 (30)	417 (58) 297 (41)	485 (84) 95 (16)	<0.0001
Viral suppression (<1000 RNA copies/ml) Yes No	715 (82) 154 (18)	335 (85) 61 (15)	380 (80) 93 (20)	0.102

Abbreviation: RDS, respondent‐driven sampling.

^a^Tabulated values may not add up to marginal values due to missing data.

### 3.2 Comparing the proportion of MSM with unsuppressed viral loads and hard‐to‐reach characteristics by recruitment strategy

There was a higher proportion of those living with HIV among participants recruited via DR (84%) compared to those recruited through RDS (58%) (*p*‐value <0.0001). Among those living with HIV and recruited through RDS, 15% were not virally suppressed. The level of unsuppressed viral loads was higher among those recruited through DR (20%); however, this was not statistically significantly different.

There were a higher proportion of participants without a high school diploma among those who were recruited through DR efforts (17%) compared to those recruited via RDS efforts (13%) (*p*‐value = 0.028). DR also resulted in a higher proportion of Black participants (75%) compared to RDS (72%) (*p*‐value <0.0001). There were no other significant differences between recruitment strategies in income level, health coverage, sexual orientation, hiding one's sexual orientation or comfort with participating in the LGBT community.

### 3.3 Trends in 'hard‐to‐reach' characteristics and unsuppressed viral loads across RDS recruitment waves

We compared proportions of socio‐demographics, HIV status and viral suppression levels across waves 0, 1, 2, 3–6 and 7–17 (Table 3). There was an increase in the proportion of those who were low income (*p* = 0.025), those with no healthcare coverage (*p* = 0.017), those who identified as bisexual (*p* < 0.0001) and those who hide their sexual orientation (*p* = 0.006) across waves of recruitment. There were decreases in the proportion of Black participants (*p* = 0.008) and those living with HIV (*p* < 0.0001), while the level of unsuppressed viral loads among those with HIV remained stable across waves (*p* = 0.08).

**Table 3:** Proportion (%) distribution of hard‐to‐reach characteristics by wave

	Wave number					
Variable (*n*)^a^	0	1	2	3–6	7–17	Trend test*p*‐value
Ethnicity						
Hispanic (*n* = 129)	12.4	15.1	17.6	10.8	11.9	0.553
Race						
Asian (*n* = 10)	0.8	0.5	1.7	1.4	1	0.008
Black (*n* = 646)	75.7	62.7	61.3	63.6	58.9	
White (*n* = 207)	12	22.7	18.5	23.8	29.7	
Other (*n* = 124)	11.6	14.1	18.5	11.2	10.4	
Education						
Less than high school (*n* = 119)	10.1	14.1	10.1	10.3	15.9	0.234
High school and beyond (*n* = 867)	89.9	86	89.9	89.7	84.1	
Income						
Low income (<$20,000) (*n* = 246)	21.8	22.1	23.5	28	29.4	0.025
Higher income (≥$20,000) (*n* = 739)	78.2	77.8	76.5	72	70.7	
Health coverage/insurance						
No (*n* = 142)	13.5	13.5	5.04	13.1	23.4	0.017
Yes (*n* = 844)	86.5	86.5	95.0	86.9	76.6	
Sexual orientation						
Other (*n* = 82)	7.5	4.9	6.7	10.8	11.0	<0.0001
Bisexual (*n* = 238)	19.9	22.7	23.5	23.4	32.3	
Gay/lesbian/homosexual (*n* = 666)	72.7	72.4	69.8	65.9	56.7	
Hides sexual orientation from other people						
Not at all (*n* = 466)	56.3	57.5	50.9	45.5	47.2	0.006
Somewhat (*n* = 345)	34	34.5	42.0	46.6	37.1	
Very much (*n* = 89)	9.7	8.1	7.14	7.9	15.7	
Participating in the LGBT community is a positive thing to do						
Agree (*n* = 525)	55.8	57.3	57.1	51.4	45.8	0.716
Neutral (*n* = 207)	18.0	21.6	23.5	20.6	23.4	
Disagree (*n* = 183)	18.7	17.8	16.0	19.6	19.4	
Prefer not to answer (*n* = 71)	7.5	3.2	3.4	8.4	11.4	
HIV status						
Negative (*n* = 352)	20.7	38.9	42.9	39.3	46.0	<0.0001
Positive (*n* = 627)	79.3	61.1	57.1	60.7	54.0	
Viral suppression						
Suppressed (*n* = 496)	79.0	81.3	81.8	91.7	81.3	0.077
Not suppressed (*n* = 104)	21.0	18.8	18.2	8.3	18.8	

^a^Sum of *n* may not be consistent due to variable specific missing data.

## 4 DISCUSSION

### 4.1 Summary of findings

In this analysis, we have compared the proportion of those who were living with HIV with unsuppressed viral loads and socio‐demographics characteristic of hard‐to‐reach populations based on recruitment methods. There was a significantly higher proportion of those screened who were living with HIV who were recruited through DR compared to RDS. The proportion of those with unsuppressed viral loads among those living with HIV was also higher among those recruited through DR; however, this difference was not statistically significant. Thus overall, RDS did not yield a higher number of MSM living with HIV with unsuppressed viral loads. Concerning socio‐demographics, those recruited via DR were more likely to be Black or have less than a high school diploma with no other differences in income level, health coverage, sexual orientation, openness of sexuality or agreement with participating in the LGBT community.

When assessing if there was an increase in 'hard‐to‐reach' characteristics across waves of RDS recruitment, there was an increase in the proportion of those who were low income, did not have health coverage, identified as bisexual and who hid their sexual orientation. However, there was a decrease in the proportion of Black participants and those living with HIV, and the proportion of unsuppressed viral loads did not increase with deeper waves.

### 4.2 Direct recruitment more efficient in identifying MSM with HIV with unsuppressed viral loads

Study findings indicate that although RDS can be successful in identifying MSM living with HIV across diverse settings, RDS did not appear to be more efficient than DR in identifying MSM living with HIV who were not virally suppressed. This is evident by the fact that although DR began 7 months into recruitment, there was a higher proportion of MSM with HIV and with unsuppressed viral loads in a shorter amount of time with DR compared to solely relying on RDS. This could be because RDS requires more time and engagement from participants to approach potential referrals rather than study staff. Though this result contrasts older findings of RDS being an efficient means of finding 'hidden' populations [26], a more recent study has also found RDS as a difficult means of finding diverse populations of MSM [27]. This study cites that relying on RDS prolonged the recruitment process as most participants did not refer future participants and ones who did only referred one participant at most. Additionally, in a 2018 analysis that supplemented random digit dialling with RDS, researchers found that RDS was helpful in increasing numbers of their target population; however, there was not enough time allotted to reach their recruitment goals [28]. They cite their failure to recruit a sufficient number of productive seeds prior to RDS as a part of the reason they did not reach recruitment targets. Thus, researchers should consider implementing DR efforts that are informed by active members of the target community prior to RDS to increase chances of recruiting productive seeds.

The clinical‐based recruitment we used during DR contributed to its success in recruiting MSM living with HIV who were not virally suppressed. For instance, study staff were able to identify MSM who were virally unsuppressed using electronic medical records, identify MSM patients who were hospitalised for serious AIDS‐related conditions and reach out to support groups that were comprised of people looking for help with ART adherence. Thus, although researchers may search outside of a clinical setting to find MSM with unsuppressed viral loads based on the assumption that these MSM are not engaged in care, we primarily found MSM who were engaged in some form of HIV care but were still not virally suppressed. This is a lesson to consider MSM who are in varying stages of the HIV care continuum when searching for those with unsuppressed viral loads.

### 4.3 Increasing waves of RDS approach hidden MSM populations

It is important to note that with deeper waves of RDS recruitment there were higher proportions of MSM who identified as bisexual and who were not comfortable with their sexuality. These men may be less likely to engage in research concerning MSM as they may fear revealing their sexuality to others when attempting to participate in research or engage in HIV care targeting MSM. This indicates that with more time and longer chains of recruitment, it is possible to tap into populations that are usually hidden in research concerning HIV care among MSM. Thus, RDS can still be useful in recruiting hard‐to‐reach populations with sufficient resources. However, researchers should consider supplementing RDS recruitment with more DR strategies when attempting to reach MSM living with HIV who are not virally suppressed because there was not an increase in this population across waves. For example, a recently developed method called 'Starfish Sampling', which utilises both venue‐based DR and peer referral sampling, has shown promise in recruiting vulnerable populations including transgender men [29].

### 4.4 Strengths and limitations

Strengths of this study include that it provides a depiction of socio‐demographics among a large, diverse sample of MSM across multiple metropolitan sites. All study sites also had community outreach programming, making it easier to find potential seeds for RDS recruitment. This programming also allowed us to accrue a sizeable number of participants through DR, making it possible to make compare participants based on recruitment strategy.

The study has several limitations. The fact that most participants with HIV were virally suppressed is encouraging; however, this finding could be credited to the fact that DR efforts in the study yielded participants from well‐established HIV health clinics and university hospitals with strong community outreach programs. The hours of study participation were limited at several sites (e.g. 9:00 AM to 2:00 PM) and were on weekdays only, potentially making it difficult for those who are most disengaged from HIV care from participating. The study sample also included a small proportion of persons who self‐identified as transgender women, many of whom were recruited through MSM social network members and not through targeted transgender outreach. Therefore, our findings cannot sufficiently speak to this small subset of the study sample. Furthermore, these results do not speak to MSM outside of the context of the United States, thus similar analyses should be repeated in other countries to determine if RDS efficiently identify MSM living with HIV with unsuppressed viral loads.

## 5 CONCLUSIONS

This study identified that although RDS can be a helpful tool in reaching MSM living with HIV, DR efforts are needed to specifically target those who are not virally suppressed. It is imperative that we prioritise strategies in engaging persons with unsuppressed viral loads in the effort to eliminate HIV infection in the future. Novel methods such as Starfish Sampling may provide a more efficient means to identify and engage those who are the most vulnerable to falling out of HIV care.

## COMPETING INTEREST

There are no conflicts of interest to declare among the authors.

## AUTHOR CONTRIBUTIONS

Chris Beyrer designed the original research study and the concept for the manuscript, provided guidance and supervision for the development of the manuscript, and assisted in revisions. Jowanna Malone conducted all analyses, coordinated with all co‐authors in manuscript development and wrote the main components of the manuscript. Stefan Baral provided expertise in RDS, contributed to network analyses and assisted in revisions. Zhe Wang assembled the dataset and conducted the data management. Carlos Del Rio, Kenneth H. Mayer, Stefan Baral, Jason Farley, Jill Stanton and Risha Irvin contributed in conducting the research of the original study and assisted in revisions. Theresa Gamble and Jill Stanton contributed to writing the methods, administering the research of the original study and assisted in revisions. James P. Hughes provided guidance in statistical analyses. Ethan Wilson contributed to data management. Oscar Guevara‐Perez, Adam Bocek, Josh Bruce and Ronald Gaston assisted with data collection. Vanessa Cummings assisted with revisions. Robert H. Remien designed the original research study and assisted with revisions. All authors have reviewed and approve the manuscript.
